# Parental spiritual and religious beliefs and behaviour data collected from the Avon Longitudinal Study of Parents and Children, 2020

**DOI:** 10.12688/wellcomeopenres.17010.1

**Published:** 2021-07-07

**Authors:** Yasmin Iles-Caven, Steven Gregory, Iain Bickerstaffe, Kate Northstone, Jean Golding

**Affiliations:** 1 Population Health Sciences, Bristol Medical School, University of Bristol, Bristol, UK, BS8 2BN, UK

**Keywords:** Religious belief, spirituality, religiosity, behaviour, ALSPAC, parent, sex differences, trends with age.

## Abstract

There are few studies that chart the ways in which the religious beliefs and practices of parents and their offspring vary over time. Even fewer can relate this to aspects of their physical and mental health or distinguish the different facets of the environment that may have influenced the development or loss of religious/spiritual belief and behaviours over time. This paper describes the recent data collection in the Avon Longitudinal Study of Parents and Children (ALSPAC) on the beliefs and behaviours of the study parents some 27-28 years after the first measures were collected. Questions that were previously administered to the mother and her partner on religion, spirituality, behaviours, and beliefs (RSBB) were repeated for the fourth time, together with enhanced data on RSBB. The new data are described and compared with previous responses. The most notable difference between the 9 year and the 2020 sweep was the increase of professed non-believers in both the mothers (17.5% vs 29.8%) and partners (31.9% vs. 45.3%).

As expected, on each occasion study partners were less likely to acknowledge RSBB compared to the study mothers. In the latest sweep, respondents were less likely to be unsure if they believed and more likely to not believe. Responses to “Do you believe in God or a divine power?” in mothers ranged from 49.9% stating ‘yes’ antenatally to 43.5% doing so in 2020; 14.9% vs 29.8% for ‘no’ and 35.2% to 26.6% for ‘not sure’. For partners, the corresponding figures are: ‘yes’ 37.0% vs. 30.0%; ‘no’ 28.6% vs. 45.3% and ‘not sure’ 34.5% vs. 24.6%. We plan to undertake detailed analyses of the antecedents and consequences of RBSS. All data are available for use by interested researchers.

## Introduction

There is increasing evidence that Western populations are becoming increasingly secular with each new generation (e.g., Chaves, 2017; Iles-Caven *et al*., 2019; Office of National Statistics, 2012; The Pew Forum on Religion and Public Life, 2017). For example, the British Social Attitudes Survey ( Curtice *et al*., 2019) has demonstrated a dramatic decline, between 1983 and 2018, in those with no religion (31% to 52%, respectively); identification with a Christian denomination (66% vs. 38%) and a steady increase in non-Christian beliefs (2% vs. 9%). Those who identified as being very or extremely religious remained similar (6% vs. 7%) but those stating they were extremely, or very non-religious increased from 14% in 1983 to 33% in 2018. Those who professed they had never believed in God rose from 13% to 26%. Among the younger generations particularly, there was an increased tolerance towards different religious or belief systems, including non-belief. At every age, more women than men were affiliated with a religion, believed in God, and attended religious services. These findings echo those found in the Avon Longitudinal Study of Parents and Children (ALSPAC) over time ( Iles-Caven *et al*., 2019).

Evidence, mainly from cross-sectional studies, has shown associations between religious/spiritual belief and positive health outcomes. These are described in brief elsewhere ( Iles-Caven *et al*., 2019). Nevertheless, definitive proof of causal consequences of belief is lacking. Any study designed to identify whether some aspect of religious/spiritual beliefs or behaviours (RSBB) affects physical or mental health must analyse data longitudinally (i.e., by identifying beliefs first and determining their relationships with subsequent aspects of health). In addition, it is also important to have information collected longitudinally on factors that may be confounders, moderators, or mediators. To this end, the Avon Longitudinal Study of Parents and Children (ALSPAC) received funding to enhance the data resource on the topic of RSBB in 2020, which will be analysed along with extensive additional data on potential mediators, moderators, confounders, and physical and mental health outcomes to be collected over the next two years.

This paper describes the RSBB data collected in 2020 from the parents enrolled ALSPAC. These questions were designed to enable linkage to other longitudinal data from the cohort on the environment, traumatic incidents, physical and mental health, and genetic background. It can be used for research into various aspects of the antecedents and consequences of RSBB, and changes over time. A separate paper will describe the RSBB data collected on the 28–29-year-old offspring in 2020.

Previous data on RSBB collected prenatally and on two subsequent occasions (at 5 and 9 years post-delivery) are described in detail elsewhere ( Iles-Caven *et al*., 2019). The data showed strong sex differences (all P<0.001) regarding RSBB (e.g., 49.9% of women vs. 37% of men stated that they believed in God/a divine power; 28.6% of men vs 14.9% of women declared they were non-believers). Among the 6256 women and 2355 men who responded at all three time points, a slight increase over time in the proportion stating that they were non-believers and a small reduction in professed belief were apparent. 

## Materials and methods

### Participants

The ALSPAC survey was specifically designed to determine ways in which the individual’s genotype combines with environmental pressures to influence health and development [ Golding *et al*., 2001]. The study is geographically based in the south-west of England, centred around the city of Bristol and its surrounding rural and semi-urban areas, with a population of about 1 million. To capture as much valid information as possible, unbiased by knowledge of details of the characteristics of the baby, the study was designed to start as early in pregnancy as possible. All women resident in the area at the time they were pregnant were eligible, provided that their expected date of delivery lay between 1 ^st^ April 1991 and 31 ^st^ December 1992. In total, 14,541 pregnant mothers, resident in the area, were recruited into the ALSPAC study. From these pregnancies, there were a total of 14,676 fetuses and 14,062 live births. Of the children, 13,988 were still alive at 1 year of age. Mothers were considered enrolled if they had returned at least one questionnaire or attended a “Children in Focus” clinic by 19 ^th^ July 1999. At the age of 7 years, the study team reached out to eligible mothers who had not been included in the study previously and thus recruited additional families to boost the number of participants. As such, from the age of 7 the total sample number is 15,454 live births, resulting in 15,589 fetuses, of which 14,901 were alive at 1 year of age ( Boyd *et al*., 2013; Fraser *et al*., 2013). 

Following advice from the ALSPAC Ethics and Law Committee, partners were recruited into the study *only* if the mothers wished them to be included. Questionnaires were sent to the mother who then passed the questionnaire on to the partner with a separate pre-paid return envelope. This method meant that ALSPAC were unable to follow up or communicate directly with the partners ( Birmingham, 2018; Fraser *et al*., 2013). Therefore, the numbers of partners’ questionnaires returned were less than those received from the mothers. Around 75% of partners participated in the study. Partners were subsequently enrolled in their own right in 2010 (n=3000).

A detailed data dictionary on the study web pages, and a detailed proposal form for access to specified data are available.

Data have been collected from pregnancy onwards using a variety of methods: (a) self-completion questionnaires; (b) assays of biological samples; (c) hands-on examination of the subjects; (d) linkage to educational and health data on the individuals; (e) linkage of addresses to measures of geographic exposures; (f) information on schools attended with details of behaviour of the child and his/her parents completed by teachers and head teachers.

Since the offspring were aged 22 years, data have been collected and managed using REDCap electronic data capture tools hosted at the University of Bristol ( Harris *et al*., 2009). REDCap (Research Electronic Data Capture) is a secure, web-based software platform designed to support data capture for research studies.

### Previous data collection on religious/spiritual beliefs and behaviour (RSBB)

The population of Avon comprises a predominantly protestant Christian population, with a lower rate of non-Christian religions (ALSPAC: 3.5% mothers; 3.5% partners) than the general UK population (6.2%). Whilst population-level religious affiliation data for England or Avon in 1991 are not available, we can illustrate the similarities between ALSPAC, the city of Bristol and England and Wales ten years later, when the national Census collected data on religion for the first time. Partners, along with the general Bristol population, were more likely to state they had no religion compared with the rest of the country ( Table 1).

**Table 1.** Comparison of the stated religious affiliation from the national census 2001 (the first year the national census collected this data), with ALSPAC data collected at the 9-year sweep (2000/2001).

Belief	ALSPAC Mothers 2000/2001	ALSPAC Partners 2000/2001	Bristol 2001 Census Men & Women	England & Wales 2001 Census Men & Women
None	15.8%	25.2%	27.0%	16.0%
Christian	80.7%	71.3%	68.5%	77.8%
All non-Christian	3.5%	3.5%	4.5%	6.2%

The initial religious behaviour and belief questions used by ALSPAC were asked at three time points (antenatally, and at 5 and 9 years later) were identical and are described elsewhere ( Iles-Caven *et al*., 2019). In brief, the items covered the following aspects of belief: (a) the participants’ fundamental beliefs: ‘Do you believe in God or some divine power?’ which had three possible responses: ‘yes; not sure; no’; (b) whether they felt that they had received or asked for assistance from such a power; (c) the type of religion the parent reported, almost all were Christians of various denominations – they were also asked how long they had had that particular faith; (d) the frequency with which they attended religious services; and (e) whether they had received help and/or support from members of their own and/or other religions. When the child was 9 years old, an additional question was asked of parents concerning whether they prayed ‘even when not in trouble’. 

Table 2 illustrates the social demographic distributions antenatally and at the 2020 sweep for each parent (age of the parents at birth, highest education level, social class (based on occupation), housing, employment, composition of the household, and ethnicity). The ages of the parents at enrolment in pregnancy ranged from 14 to 47 years, median 28, for mothers, and from 15 to 70, median 30 for partners. The respondents to the 2020 questionnaires were proportionately older (mothers aged 43 to 76; partners aged 44 to 99), better educated, more likely to have lived with their partner during pregnancy and less likely to have non-White ethnic backgrounds (this was especially true of the partners).

**Table 2.** Proportion (n) of enrolled parents who completed the religion questions in a) pregnancy (1991–2) and in b) 2020 by selected sociodemographic factors.

	Mothers in Pregnancy	Mothers 2020	Partners in Pregnancy	Partners 2020
*Age of parents at the birth*				
<25	21.2% (2599)	12.6% (571)	21.3% (2042)	15.5% (364)
25–34	68.5% (8384)	74.2% (3359)	68.4% (6553)	58.4% (1371)
35+	10.3% (1260)	12.4% (595)	10.3% (988)	26.1% (614)
Highest parental education level *				
Low (<O level)	28.5% (3304)	16.4% (714)	26.9% (2436)	11.1% (219)
Medium (O level)	35.2% (4089)	34.2% (1505)	34.9% (3154)	20.5% (406)
High (>O level)	36.3% (4219)	49.4% (2174)	38.2% (3450)	68.4% (1353)
*Partner lived with mother during* *pregnancy*				
Yes	91.7% (11109)	96.4% (4180)	95.2% (9018)	98.1% (2014)
No	8.3% (1003)	3.6% (154)	4.8% (456)	1.9% (39)
*Sex of child*				
Boy	51.5% (6323)	47.3% (2262)	51.5% (4949)	46.7% (1022)
Girl	48.5% (5950)	51.8% (2518)	48.5% (4670)	53.3% (1176)
*Ethnic* * background*				
White	97.6% (11288)	98.0% (4291)	97.2% (9367)	98.3% (2019)
Other than White	2.4% (273)	2.0% (89)	2.8% (268)	1.7% (35)

*Public exams, usually in 5–10 subjects, are normally undertaken at the end of Year 11 (age 16, although they can be taken at any age). Formerly called ‘O’ (Ordinary) Levels the current equivalent are GCSEs.

### Enhanced data collection on RSBB

The religion questions used at the three earlier time points were repeated in the 2020 sweep (see Table 3, questions C1-C6, C9, C10), and enhanced with additional questions ( Table 3: C7, C8, C11-C24, highlighted in bold) to capture details of extrinsic/intrinsic behaviour, and interests in religious worship using the written word and/or radio, television or other electronic media. Most of these new questions comprised elements from well-validated, standardised scales recommended by an international workshop of RSBB/Health experts in August 2019 (see Acknowledgements) and are described below. In the 2020 sweep, both the parents and their offspring received identical RSBB questions. 

**Table 3.** Questions (numbered as in the questionnaire) asked of the mother and partners 27+ years post-delivery (2020) with their variable names (mothers have suffix _M, partners _P and corresponding variable numbers (e.g., Y3000_M) and number of valid responses. Questions not previously asked of the cohort are in bold.

Questions 2020	Variable	Mothers valid responses (Total 4663)	Partners valid responses (Total 2181)
*C1. Do you believe in God or in some divine power?* Yes/Not sure/No	*Y3000*	4627	2157
*C2. Do you feel that God (or some divine power) has helped you at any time? * Yes/Not sure/No	*Y3010*	4616	2144
*C3. Would you appeal to God for help if you were in trouble? * Yes/Not sure/No	*Y3020*	4611	2147
*C4. Do you ‘pray’ even if not in trouble? * Yes/No	*Y3030*	4585	2143
*C5. What sort of religious faith would you say you had? (tick only one)* None; Church of England; Roman Catholic; Jehovah’s Witness; Christian Science; Mormon; Other Christian (please describe); Jewish; Buddhist; Sikh; Hindu; Muslim; Rastafarian; Other (please describe)	*Y3040*	4578	2125
*C6. How long have you had this particular faith?* All my life/More than 5 years/3-5 years/ 1-2 years/ Less than a year	*Y3050*	4548	2107
***C7. Were you brought up in this faith?*** Yes/No/If no, please describe what faith if any	*Y3060*	4487	2094
*C8.* Did you bring your child(ren) up in your current faith/belief (including none)? Yes this faith/No. If no, what faith did you bring your children up in, if any?	*Y3070*	4536	2111
*C9. How often do you go to a place of worship or other religious meetings?* Yes, at least once a week/Yes, at least once/month/Yes, at least once/year/Not at all	*Y3080*	4579	2139
*C10. Do you obtain help and support:*			
*-From leaders of your religious group? * Yes/No	*Y3090*	4556	2132
*-From other members of your religious group?* Yes/No	*Y3091*	4507	2106
*-From leaders of other religious groups (please describe)*? Yes/No	*Y3092*	4294	2019
*-From members of other religious groups (please describe)*? Yes/No	*Y3093*	4247	1238
***C11. How often do you spend time in private religious activities, such as*** ***prayer, meditation, or holy scripture study?*** More than once/day/Daily/2+times/week/Once/week/ Few times/month/Rarely or never	*Y3100*	4553	2110
***C12. How often do you listen to/watch religious programming on the radio/*** *** television/social media?*** Daily/Several times/week/Several times/month/Occasionally/ Never/Please describe	*Y3110*	4587	2144
***C13. How often do you read religious related texts or publications (e.g.*** *** the Bible, the Koran, prayer book, Watchtower, The War Cry, The Friend, *** ***Spirituality & Health, Catholic Digest)*** Daily/Several times/week/Several times/month/Occasionally/ Never/Please describe	*Y3120*	4586	2147
***C14. In my life, I experience the Presence of the Divine (e.g. God)*** Definitely true of me/Tends to be true of me/Unsure/ Tends not to be true of me/Definitely not true of me/ Not applicable	*Y3130*	4551	2137
***C15. My religious beliefs are what really lie behind my whole approach to life*** Definitely true of me/Tends to be true of me/Unsure/ Tends not to be true of me/Definitely not true of me/ Not applicable	*Y3140*	4544	2135
***C16. I try hard to carry my religion over into all other dealings in life.*** Definitely true of me/Tends to be true of me/Unsure/ Tends not to be true of me/Definitely not true of me/ Not applicable	*Y3150*	4530	2131
***C17. I attend a place of worship mainly because it helps me make friends:*** Strongly agree/Mildly agree/Not sure/Mildly disagree/Strongly disagree/Not applicable	*Y3160*	4536	2131
***C18. I pray mainly to gain relief and protection.*** Strongly agree/Mildly agree/Not sure/Mildly disagree/Strongly disagree/Not applicable	*Y3170*	4521	2130
***C19. Did you ever have a religious or spiritual experience that changed your*** ***life?*** Yes/No, If yes, age/please describe	*Y3180*	4558	2140
***C20. Have you ever had a significant gain in your faith?*** Yes/No, If yes, age/please describe	*Y3190*	4525	2129
***C21. Have you ever had a significant loss of faith?*** Yes/No *,* If yes, age/please describe	*Y3200*	4532	2132
***C22. To what extent do you consider yourself a religious person?*** Very/Moderately/Slightly/Not at all	*Y3210*	4569	2144
***C23. To what extent do you consider yourself a spiritual person?*** Very/Moderately/Slightly/Not at all	*Y3220*	4569	2138
***C24. How important to you is religion or spirituality?*** Highly/Moderately/Slightly/Not important at all	*Y3230*	4570	2146

### The new measures

The Duke University Religion Scale (DUREL) ( Koenig *et al*., 1997), a five-item measure of religious involvement was developed for use in large cross-sectional or longitudinal studies. It assesses organisational and non-organisational religious activity and intrinsic religiosity. The scale has high test-retest reliability (intra-class correlation = 0.91), high internal consistency (Cronbach’s alpha’s = 0.78-0.91); and high convergent validity with other religiosity measures (r’s = 0.71-0.86). The DUREL has been used extensively ( Koenig *et al*., 2011). The five questions that comprised this scale were split up in the questionnaire (see Table 3, questions C9, C11, C14-C16). As can be seen from Table 4, women were more likely than their partners to attend organised religious worship and to practice private worship (e.g., prayer). Questions C9 and C11 measure organisational activity; and questions C14-C16 measure intrinsic religious motivation when combined, and again the women scored higher (means 6.44 vs. 5.52).

**Table 4.** Duke University Religion Scale (DUREL) derived variables.

		Mothers	Partners
	**Organised religion activity score**		
1	Not at all	2359 (49.8%)	1254 (58.0%)
2	Occasionally	1388 (29.3%)	494 (22.9%)
3	At least 1/yr	359 (7.6%)	162 (7.5%)
4	At least 1/mth	205 (4.3%)	83 (3.8%)
5	1+/week	423 (8.9%)	168 (7.8%)
	**Private religious activity score**		
1	Rarely	3535 (75.1%)	1768 (83.0%)
2	Few/month	287 (6.1%)	84 (3.9%)
3	1/wk	129 (2.7%)	38 (1.8%)
4	2+/wk	270 (5.7%)	80 (3.8%)
5	Daily	354 (7.5%)	100 (4.7%)
6	>1/day	130 (2.8%)	60 (2.8%)
	**Intrinsic score**	4664 (N)	2143 (N)
	Mean	6.44	5.52
	SD	4.04	3.74
	Range	3–15	3–15
	**DUREL Total Index**	4576 (N)	2090 (N)
	Mean	10.15	8.89
	SD	6.11	5.72
	Range	5–26	5–26

SD = standard deviation.

Specific questions to elicit extrinsic and intrinsic religious motivation were included for the first time. Extrinsic individuals are more likely to exploit religion, e.g., to provide security and solace, for social reasons, status, and self-justification. Whereas intrinsic individuals aim to live their life according to the tenets of that religion and exhibit behaviours consistent with those tenets. Of the original Allport & Ross scale (1967), 14 questions had been selected and revised by Gorsuch & McPherson (1989) so that the questions could be answered by non-believers. Each question has a five-point scale ranging from ‘strongly agree’ to ‘strongly disagree’, with an additional ‘not applicable’ option. We used two of the extrinsically weighted items (see Table 3, questions C17 and C18).

Three questions (see Table 3, questions C19-C23) are from the well-validated Fetzer Brief Multi-Dimensional Measure of Religiosity/Spirituality for use in health research (BMMRS) ( Fetzer Institute, 2003). The questions were chosen to enquire about religious/spiritual history: whether an individual has had a religious/spiritual experience that changed their life or experienced a significant gain or loss of faith and if so when. Note that the study team added a request for a free text description of the experience/gain/loss in faith after discussion with the workshop attendees. This request was worded in such a way that it was seen as optional by the respondents. 

At the suggestion of Connie Svob (personal communication) question C24, “How important to you is religion or spirituality?” was included. This question has been shown to be highly predictive in a transgenerational longitudinal study of depressed and nondepressed probands and their offspring followed over 30 years ( Anderson *et al*., 2021; Weissman *et al*., 2016). Future comparison with the ALSPAC data was thought to be of great value.

Questions C7 and C8 were devised by the study team and asked whether the participants were brought up in a particular faith and whether they had brought their own child(ren) up in a particular faith.

Questions C12 and C13 were devised by Golding & Iles-Caven for this sweep to elicit contemporary forms of private belief/worship such as radio, TV programmes and social media, and the reading of religious texts and periodicals.

In each of the questionnaires administered at the four time points (pregnancy; +5years; +9 years, and in 2020) the question (C6) concerning the duration of their current faith was included. In pregnancy, the majority responded: ‘all my life’ and fewer than 5% responded <5 years. These responses were consistent over time and enable the study to identify a large proportion of the population for whom there are consistent responses throughout the time span (see below and Table 8a, Table 8b for examples). We believe that using this data to extrapolate backwards for this large group of the population is valid, especially when supported by earlier data. We can identify a large sub-group for whom data on RSBB will be able to be extrapolated throughout the life-course ( Table 5). 

**Table 5.** Depiction of data collected on RSBB at various stages (years from birth of the index offspring) for mothers and their partners, where + indicates actual data collection, and E denotes extrapolation backwards.

Time	PP	P	0-1	2-3	4-5	6-7	8-9	10-11	12-13	14-15	16-17	18-19	20-21	22-23	24-26	27-28
RSBB	E	+	E	E	+	E	+	E	E	E	E	E	E	E	E	**++**

RSBB = religion, spirituality, behaviours, and beliefs, PP = pre-pregnancy; P = during pregnancy; ++ = RSBB data collected in 2020

Table 6 shows the mothers’ and partners’ response rates to each question in the 2020 sweep. There were many differences evident between the sexes (at p<0.001 level). Women were more likely than men to believe in a divinity, participate in private and public worship, and to lead their lives according to their religious principles.

**Table 6.** Parental responses to each question in 2020 (p values compare the responses between mother and partner).

Question	Mothers No. (%)	Partners No. (%)	P value
*Do you believe in God or some divine power?*			
Yes	2082 (43.5)	654 (30.0)	<0.001
Not sure	1429 (29.9)	538 (24.7)	
No	1270 (26.6)	986 (45.3)	
*Do you believe that God/divine power has helped you at any time?*			
Yes	1651 (34.6)	509 (23.5)	<0.001
Not sure	1222 (25.6)	424 (19.6)	
No	1897 (39.8)	1233 (56.9)	
*Would you appeal to God for help if you were in trouble?*			
Yes	2319 (48.7)	670 (30.9)	<0.001
Not sure	937 (19.7)	410 (18.9)	
No	1510 (31.7)	1089 (50.2)	
*Do you ‘pray’ even if not in trouble?*			
Yes	1602 (33.8)	448 (20.7)	<0.001
Not sure	328 (6.9)	129 (6.0)	
No	2809 (59.3)	1588 (73.3)	
*Did you bring your child(ren) up in your current faith/belief (including none)?* *If no, what faith did you bring your children up in, if any?*			
Yes, this faith	3177 (67.6)	1335 (62.7)	<0.001
No	1524 (32.4)	794 (37.3)	
*How long have you had this particular faith?*			
Whole life	3467 (74.8)	1434 (67.8)	<0.001
>5 years	1091 (23.5)	649 (30.7)	
3–5 years	46 (1.0)	23 (1.1)	
<3 years	34 (0.7)	10 (0.5)	
*Do you go to a place of worship?*			
At least once a week	423 (8.9)	168 (7.8)	<0.001
At least once a month	205 (4.3)	83 (3.8)	
At least once a year	359 (7.6)	162 (7.5)	
Occasionally	1388 (29.3)	494 (22.9)	
Never	2359 (49.8)	1254 (58.0)	
*Do you obtain help and support:*			
* From leaders of your religious group?*			
Yes	431 (9.2)	180 (8.4)	0.336
No	3161 (67.1)	1303 (60.5)	
Not applicable *	1117 (23.7)	671 (31.2)	
* From members of your religious group?*			
Yes	536 (11.5)	203 (9.5)	0.021
No	3012 (64.7)	1254 (58.9)	
Not applicable *	1110 (23.8)	671 (31.5)	
* From leaders of other religious group?*			
Yes	68 (1.5)	31 (1.5)	0.813
No	4373 (98.5)	2009 (98.5)	
* From members of other religious groups?*			
Yes	110 (2.5)	46 (2.3)	0.378
No	4283 (97.5)	1972 (97.7)	
*Type of religious belief*			
Stated “none”	1285 (27.2)	864 (40.2)	<0.001
Church of England	2313 (48.9)	889 (41.4)	
Roman Catholic	361 (7.6)	137 (6.4)	
Jehovah’s Witness	20 (0.4)	8 (0.4)	
Methodist	182 (3.8)	57 (2.7)	
Baptist/Evangelical	171 (3.6)	56 (2.6)	
Other Christian (please describe) *	126 (2.7)	52 (2.4)	
Judaism, Sikh, Hinduism, Muslim	27 (0.5)	14 (0.7)	
Buddhist	34 (0.7)	17 (0.8)	
Other non-Christian	213 (4.5)	53 (2.5)	
***How often do you spend time in private religious activities, such as prayer,*** ***meditation, or holy scripture study?***			
More than once/day	130 (2.8)	60 (2.8)	<0.001
Daily	354 (7.5)	100 (4.7)	
2+ times/week	270 (5.7)	80 (3.8)	
Once/week	129 (2.7)	38 (1.8)	
Few times/month	287 (6.1)	84 (3.9)	
Rarely or never	3535 (75.1)	1768 (83.0)	
***How often do you listen to/watch religious programming on the radio/*** *** television/social media?***			
Daily	41 (0.9)	19 (0.9)	<0.001
Several times/week	91 (1.9)	33 (1.5)	
Several times/month	129 (2.7)	47 (2.2)	
Occasionally	1421 (30.0)	556 (25.7)	
Never	3059 (64.5)	1511 (69.8)	
***How often do you read religious related texts or publications (e.g. the Bible, the Koran,*** *** prayer book, Watchtower, The War Cry, The Friend, Spirituality & Health, Catholic Digest)***			
Daily	214 (4.5)	78 (3.6)	0.05
Several times/week	123 (2.6)	47 (2.2)	
Several times/month	106 (2.2)	49 (2.3)	
Occasionally	641 (13.5)	256 (11.8)	
Never	3656 (77.1)	1739 (80.2)	
***In my life, I experience the Presence of the Divine (e.g. God)***			
Definitely, true of me	503 (10.7)	161 (7.5)	<0.001
Tends to be true of me	507 (10.8)	156 (7.2)	
Unsure	793 (16.9)	264 (12.2)	
Tends not to be true of me	411 (8.7)	166 (7.7)	
Definitely, not true of me	1345 (28.6)	819 (37.9)	
Not applicable *	1146 (24.4)	593 (27.5)	
***My religious beliefs are what really lie behind my whole approach to life***			
Definitely true of me	461 (9.8)	157 (7.3)	<0.001
Tends to be true of me	723 (15.4)	257 (11.9)	
Unsure	520 (11.1)	150 (7.0)	
Tends not to be true of me	491 (10.5)	195 (9.0)	
Definitely not true of me	1256 (26.7)	693 (32.1)	
Not applicable *	1247 (26.5)	704 (32.7)	
***I try hard to carry my religion over into all other dealings in life.***			
Definitely true of me	411 (8.8)	152 (7.1)	<0.001
Tends to be true of me	667 (14.2)	213 (9.9)	
Unsure	500 (10.7)	156 (7.2)	
Tends not to be true of me	454 (9.7)	168 (7.8)	
Definitely not true of me	1296 (27.7)	702 (32.6)	
Not applicable *	1356 (28.9)	762 (35.4)	
***I attend a place of worship mainly because it helps me make friends:***			
Strongly agree	87 (1.9)	18 (0.8)	0.014
Mildly agree	352 (7.5)	127 (5.9)	
Not sure	155 (3.3)	80 (3.7)	
Mildly disagree	309 (6.6)	123 (5.7)	
Strongly disagree	939 (20.0)	406 (18.9)	
Not applicable *	2847 (60.7)	1399 (65.0)	
***I pray mainly to gain relief and protection.***			
Strongly agree	212 (4.5)	39 (1.8)	<0.001
Mildly agree	748 (16.0)	178 (8.3)	
Not sure	388 (8.3)	123 (5.7)	
Mildly disagree	351 (7.5)	146 (6.8)	
Strongly disagree	740 (15.8)	369 (17.1)	
Not applicable *	2235 (47.8)	1297 (60.3)	
***Did you ever have a religious or spiritual experience that changed your life?***			
Yes	542 (11.5)	196 (9.1)	0.003
No	4171 (88.5)	1966 (90.9)	
***Have you ever had a significant gain in your faith?***			
Yes	481 (10.3)	184 (8.6)	0.025
No	4199 (89.7)	1967 (91.4)	
***Have you ever had a significant loss of faith?***			
Yes	660 (14.1)	290 (13.5)	0.582
No	4024 (85.9)	1864 (86.5)	
***To what extent do you consider yourself a religious person?***			
Very	113 (2.4)	46 (2.1)	<0.001
Moderately	653 (13.8)	255 (11.8)	
Slightly	1549 (32.8)	512 (23.6)	
Not at all	2408 (51.0)	1352 (62.4)	
***To what extent do you consider yourself a spiritual person?***			
Very	368 (7.8)	99 (4.6)	<0.001
Moderately	937 (19.8)	347 (16.1)	
Slightly	1400 (29.6)	439 (20.3)	
Not at all	2019 (42.7)	1275 (59.0)	
***How important to you is religion or spirituality?***			
Highly important	662 (14.0)	222 (10.2)	<0.001
Moderately important	793 (16.8)	284 (13.1)	
Slightly important	1417 (30.0)	476 (22.0)	
Not important at all	1853 (39.2)	1186 (54.7)

*P values are calculated excluding the ‘not applicable’ responses.

Table 7a and Table 7b show the total numbers of each parent who answered the RSBB questions at *any* of the four time points of data collection, and Table 8a and Table 8b show only those parents who completed the questions at *every* sweep. Modern statistical techniques will allow imputation increasing statistical power for those for whom we have incomplete data.

**Table 7a.** Mother’s beliefs/religion and support at each time point, where data for the questions are available.

Question	Antenatal	5 years	9 years	2020
	N	N	N	N
*Do you believe in God or some divine power?*				
Yes	6160 (49.9%)	4141 (46.5%)	3776 (48.2%)	2016 (43.6%)
Not sure	4353 (35.2%)	3018 (33.9%)	2682 (34.3%)	1231 (26.6%)
No	1838 (14.9%)	1745 (19.6%)	1369 (17.5%)	1380 (29.8%)
*Do you believe that God/divine power has helped you at any* * time?*				
Yes	4181 (33.9%)	2672 (30.1%)	2566 (32.9%)	1598 (34.6%)
Not sure	4672 (37.9%)	3047 (34.3%)	2774 (35.6%)	1184 (25.6%)
No	3477 (28.2%)	3152 (35.5%)	2454 (31.5%)	1834 (39.7%)
*Would you appeal to God for help if you were in trouble?*				
Yes	5738 (46.6%)	4070 (45.9%)	3578 (45.8%)	2241 (48.6%)
Not sure	3861 (31.3%)	2653 (29.9%)	2288 (29.3%)	903 (19.6%)
No	2722 (22.1%)	2146 (24.2%)	1943 (24.9%)	1467 (31.8%)
*Mother prays even if not in trouble*				
Yes	-	-	3012 (39.2%)	1552 (33.8%)
No	-	-	4677 (60.8%)	2715 (59.2%)
Not sure *			-	318 (6.9%)
*Mother brought up child in this faith* *including none*				
Yes	-	-	5167 (72.0%)	3074 (67.6%)
No	-	-	2010 (28.0%)	1474 (32.4%)
*Length of time mother has followed her current religion*				
Whole life	8905 (81.8%)	6610 (83.6%)	5667 (80.8%)	3365 (75.0%)
>5 years	1472 (13.5%)	1018 (12.9%)	1135 (16.2%)	1047 (23.3%)
3–5 years	290 (2.7%)	147 (1.9%)	119 (1.7%)	43 (1.0%)
<2 years	215 (2.0%)	127 (1.7%)	90 (1.3%)	32 (0.7%)
*Frequency mother attends a place of* * worship*				
At least once a week	885 (7.3%)	886 (10.3%)	927 (12.0%)	405 (8.8%)
At least once a month	836 (6.9%)	849 (9.8%)	723 (9.4%)	200 (4.4%)
At least once a year	3520 (29.2%)	2287 (26.5%)	2276 (26.4%)	1697 (37.1%)
Never	6824 (56.6%)	4602 (53.4%)	3838 (49.7%)	2277 (49.7%)
*Has the mother received help from:*				
* Leaders in her religious group*				
Yes	897 (7.7%)	645 (7.6%)	738 (10.0%)	413 (9.1%)
No	10735(92.3%)	7789(92.4%)	6620 (90.0%)	4143 (90.9%)
* Members of her religious group*				
Yes	1087(9.4%)	856 (10.2%)	921 (12.6%)	513 (11.4%)
No	10465(90.6%)	7499 (89.8%)	6384 (87.4%)	3994 (88.6%)
* Members of other religious groups*				
Yes	233(2.1%)	144 (1.8%)	186 (2.6%)	107 (2.5%)
No	11059(97.9%)	7911 (98.2%)	6862 (97.4%)	4140 (97.5%)
*Type of religious belief*				
Stated “none”	1979 (16.3%)	1408 (16.2%)	1276 (16.7%)	1235 (27.0%)
Church of England	7767 (63.9%)	5528 (63.6%)	4602 (60.4%)	2238 (48.9%)
Roman Catholic	971 (8.0%)	669 (7.7%)	582 (7.6%)	350 (7.6%)
Other Christian (please describe) **	956 (7.9%)	786 (9.0%)	895 (11.7%)	486 (10.6%)
Other non-Christian (please describe) ***	474 (3.9%)	300 (3.5%)	268 (3.5%)	269 (5.9%)

*Not sure option added to 2020 sweep only **Other Christian comprises: Christian Science, Mormon, Baptists, Evangelical, Methodists, Orthodox, Jehovah’s Witness etc. ***Other non-Christian comprises: Buddhism, Judaism, Sikhism, Hinduism, Muslim, Rastafarian, Spiritualism, New Age etc.

**Table 7b.** Partner’s beliefs/religion and support at each time point, where data for the questions are available.

Question	Antenatal	5 years	9 years	2020
*Do you believe in God or some divine power?*				
Yes	3621 (37.0%)	1505 (33.6%)	1275 (35.3%)	648 (30.0%)
Not sure	3376 (34.5%)	1573 (35.1%)	1183 (32.8%)	531 (24.6%)
No	2801 (28.6%)	1406 (31.4%)	1149 (31.9%)	978 (45.3%)
*Do you believe that God/divine power has helped you* * at any time?*				
Yes	2472 (25.3%)	1031 (23.0%)	876 (24.3%)	501 (23.4%)
Not sure	3158 (32.3%)	1430 (32.0%)	1117 (31.0%)	419 (19.5%)
No	4144 (42.4%)	2013 (45.0%)	1606 (44.6%)	1224 (57.1%)
*Would you appeal to God for help if you were in* * trouble?*				
Yes	3536 (36.2%)	1586 (35.5%)	1248 (34.9%)	660 (30.7%)
Not sure	6288 (27.5%)	1319 (29.5%)	1014 (28.3%)	407 (19.0%)
No	3548 (36.3%)	1566 (35.0%)	1319 (36.8%)	1080 (50.3%)
*Father prays even if not in trouble*				
Yes	-	-	902 (25.4%)	440 (20.5%)
No	-	-	2650 (74.6%)	1575 (73.5%)
Not sure *	-	-	-	128 (6.0%)
*Father brought up child in this faith* * including none*				
Yes	-	-	2012 (60.7%)	1321 (62.7%)
No	-	-	1301 (39.3%)	786 (37.3%)
*Length of time father has followed his current religion*				
Whole life	6671 (79.0%)	3052 (78.3%)	2449 (76.2%)	1418 (67.7%)
>5 years	1409 (16.7%)	744 (19.1%)	678 (21.1%)	643 (30.7%)
3–5 years	180 (2.1%)	48 (1.2%)	54 (1.7%)	23 (1.1%)
≤2 years	183 (2.2%)	52 (1.3%)	35 (1.1%)	10 (0.5%)
*Frequency father attends a place of worship*				
At least once a week	588 (6.1%)	358 (8.2%)	322 (9.0%)	166 (7.8%)
At least once a month	415 (4.3%)	282 (6.5%)	240 (6.7%)	81 (3.8%)
At least once a year	2515 (26.2%)	987 (22.7%)	952 (26.7%)	650 (30.4%)
Never	6077 (63.3%)	2712 (62.5%)	2049 (57.5%)	1242 (59.1%)
Father receives help from:				
* Leaders in his religious group*				
Yes	559 (6.0%)	301 (7.1%)	287 (8.2%)	178 (8.3%)
No	8717 (94.0%)	3947 (92.9%)	3198 (91.8%)	1954 (91.7%)
* Members of his religious group*				
Yes	642 (7.0%)	335 (7.9%)	327 (9.4%)	200 (9.5%)
No	8544 (93.0%)	3894 (92.1%)	3146 (90.6%)	1906 (90.5%)
* Members of other religious groups*				
Yes	144 (1.6%)	65 (1.6%)	55 (1.6%)	43 (2.2%)
No	8944 (98.4%)	4093 (98.4%)	3356 (98.4%)	1954 (97.8%)
*Type of religious belief*				
Stated “none”	2633 (27.3%)	1118 (25.6%)	921 (26.3%)	859 (40.4%)
Church of England	5237 (54.3%)	2453 (56.2%)	1847 (52.8%)	876 (41.2%)
Roman Catholic	699 (7.3%)	314 (7.2%)	274 (7.8%)	135 (6.4%)
Other Christian (please describe) **	633 (6.6%)	347 (8.0%)	344 (9.8%)	52 (2.4%)
Other Non-Christian ***	437 (4.5%)	132 (3.0%)	110 (3.1%)	53 (2.5%)

*Not sure option added to 2020 sweep only **Other Christian comprises: Christian Science, Mormon, Baptists, Evangelical, Methodists, Orthodox, Jehovah’s Witness etc. ***Other non-Christian comprises: Buddhism, Judaism, Sikhism, Hinduism, Muslim, Rastafarian, Spiritualism, New Age etc.

**Table 8a.** Mother’s beliefs/religion and support at each time point, where data for the questions are available for mothers who completed all questions at all time points (N = 2042).

Question	Antenatal	5 years	9 years	2020
*Do you believe in God or some divine power?*				
Yes	1251(61.3%)	1144(56.0%)	1129(55.3%)	979(47.9%)
Not sure	657(32.2%)	695(34.0%)	712(34.9%)	663(32.5%)
No	134(6.6%)	203(10.0%)	201(9.8%)	400(19.6%)
*Do you believe that God/divine power has helped you at any time?*				
Yes	798(39.1%)	690(33.8%)	721(35.3%)	756(37.0%)
Not sure	851(41.7%)	797(39.0%)	798(39.1%)	574(28.1%)
No	393(19.2%)	555(27.2%)	523(25.6%)	712(34.9%)
*Would you appeal to God for help if you were in trouble?*				
Yes	1168(57.2%)	1112(54.5%)	1108(54.3%)	1082(53.0%)
Not sure	612(30.0%)	602(29.5%)	591(28.9%)	429(21.0%)
No	262(12.8%)	328(16.1%)	343(16.8%)	531(26.0%)
*Mother prays even if not in trouble*				
Yes	-	-	917(44.9%)	722(35.4%)
No	-	-	1125(55.1%)	1161(56.9%)
Not sure *			-	159(7.8%)
*Mother brought up child in this faith including none*				
Yes	-	-	1578(77.3%)	1457(71.4%)
No	-	-	464(22.7%)	585(28.6%)
*Length of time mother has followed her current religion*				
Whole life	1666(81.6%)	1703(83.4%)	1692(82.9%)	1569(76.8%)
>5 years	316(15.5%)	281(13.8%)	299(14.6%)	436(21.4%)
3-5 years	35(1.7%)	39(1.9%)	31(1.5%)	24(1.2%)
≤2 years	25(1.2%)	19(0.9%)	20(1.0%)	13(0.6%)
*Frequency mother attends a place of worship*				
At least once a week	187(9.2%)	251(12.3%)	273(13.4%)	173(8.5%)
At least once a month	193(9.5%)	268(13.1%)	217(10.6%)	93(4.6%)
At least once a year	798(39.1%)	717(35.1%)	774(37.9%)	181(8.9%)
Occasionally *	-	-	-	680(33.3%)
Never	864(42.3%)	806(39.5%)	778(38.1%)	915(44.8%)
Has the mother received help from:				
* Leaders in her religious group*				
Yes	187(9.2%)	177(8.7%)	220(10.8%)	175(8.6%)
No	1855(90.8%)	1865(91.3%)	1822(89.2%)	1867(91.4%)
* Members of her religious group*				
Yes	235(11.5%)	236(11.6%)	257(12.6%)	219(10.7%)
No	1807(88.5%)	1806(88.4%)	1785(87.4%)	1823(89.3%)
* Members of other religious groups*				
Yes	40(2.0%)	42(2.1%)	54(2.6%)	42(2.1%)
No	2002(98.0%)	2000(97.9%)	1988(97.4%)	2000(97.9%)
*Type of religious belief*				
Stated “none”	136(6.7%)	145(7.1%)	155(7.6%)	404(19.8%)
Church of England/ Anglican	1476(72.3%)	1455(71.3%)	1403(68.7%)	1170(57.3%)
Roman Catholic	185 (9.1%)	179(8.8%)	177(8.7%)	162(7.9%)
Other Christian (please describe) **	189 (9.3%)	229(11.2%)	260(12.7%)	211(10.3%)
Other (please describe) ***	56 (2.7%)	34(1.7%)	47(2.3%)	95(4.7%)

*’Not sure’ and ‘Occasionally’ options added to 2020 sweep only **’Other Christian’ comprises: Christian Science, Mormon, Baptists, Evangelical, Methodists, Orthodox, Jehovah’s Witness etc. ***’Other’ comprises: Buddhism, Judaism, Sikhism, Hinduism, Muslim, Rastafarian, Spiritualism, New Age etc.

**Table 8b.** Father’s beliefs/religion and support at each time point, where data for the questions are available for all partners who completed all questions at all time points (N=718).

Question	Antenatal	5 years	9 years	2020
*Do you believe in God or some divine power?*				
Yes	349(48.6%)	320(44.6%)	328(45.7%)	266(37.0%)
Not sure	245(34.1%)	263(36.6%)	243(33.8%)	193(26.9%)
No	124(17.3%)	135(18.8%)	147(20.5%)	259(36.1%)
*Do you believe that God/divine power has helped you at any time?*				
Yes	229(31.9%)	224(31.2%)	220(30.6%)	198(27.6%)
Not sure	254(35.4%)	237(33.0%)	252(35.1%)	161(22.4%)
No	235(32.7%)	257(35.8%)	246(34.3%)	359(50.0%)
*Would you appeal to God for help if you were in trouble?*				
Yes	357(49.7%)	343(47.8%)	313(43.6%)	266(37.0%)
Not sure	175(24.4%)	193(26.9%)	208(29.0%)	150(20.9%)
No	186(25.9%)	182(25.3%)	197(27.4%)	302(42.1%)
*Father prays even if not in trouble*				
Yes	-	-	238(33.1%)	179(24.9%)
No	-	-	480(66.9%)	490(68.2%)
Not sure *	-	-	-	49(6.8%)
*Father bringing up child in this faith*				
Yes	-	-	493(68.7%)	488(68.0%)
No	-	-	225(31.3%)	230(32.0%)
*Length of time father has followed his current religion*				
Whole life	528(73.5%)	537(74.8%)	540(75.2%)	490(68.2%)
>5 years	168(23.4%)	172(24.0%)	156(21.7%)	220(30.6%)
3–5 years	12(1.7%)	5(0.7%)	15(2.1%)	7(1.0%)
≤2	10(1.4%)	4(0.6%)	7(1.0%)	1(0.1%)
*Frequency father attends a place of worship*				
At least once a week	78(10.9%)	90(12.5%)	103(14.3%)	66(9.2%)
At least once a month	54(7.5%)	83(11.6%)	69(9.6%)	34(4.7%)
At least once a year	269(37.5%)	212(29.5%)	236(32.9%)	58(8.1%)
Occasionally *	-	-	-	167(23.3%)
Never	317(44.2%)	333(46.4%)	310(43.2%)	393(54.7%)
Father receives help from:				
* Leaders in his religious group*				
Yes	71(9.9%)	73(10.2%)	82(11.4%)	69(9.6%)
No	647(90.1%)	645(89.8%)	636(88.6%)	649(90.4%)
* Members of his religious group*				
Yes	90(12.5%)	82(11.4%)	98(13.6%)	81(11.3%)
No	628(87.5%)	636(88.6%)	620(86.4%)	637(88.7%)
* Members of other religious groups*				
Yes	21(2.9%)	19(2.6%)	11(1.5%)	12(1.7%)
No	697(97.1%)	699(97.4%)	707(98.5%)	706(98.3%)
*Type of religious belief*				
Stated “none”	117(16.3%)	114(15.9%)	120(16.7%)	222(30.9%)
Church of England	423(58.9%)	434(60.4%)	420(58.5%)	355(49.4%)
Roman Catholic	64(8.9%)	64(8.9%)	66(9.2%)	55(7.7%)
Other Christian (please describe) **	75(10.4%)	90(12.5%)	84(11.7%)	60(8.4%)
Other (please describe) ***	39(5.4%)	16(2.2%)	28(3.9%)	26(3.6%)

*’Not sure’ and ‘Occasionally’ options added to 2020 sweep only **’Other Christian’ comprises: Christian Science, Mormon, Baptists, Evangelical, Methodists, Orthodox, Jehovah’s Witness etc. ***‘Other’ comprises: Buddhism, Judaism, Sikhism, Hinduism, Muslim, Rastafarian, Spiritualism, New Age etc.

In the 20 years since the questions were previously asked at 9 years on belief in God/a divine power, whether God/a divine power had helped them or if they would appeal for help from such powers, mothers seem to have become more certain of their beliefs, with the largest reduction in the ‘not sure’ categories. Very few stated they had only followed their current faith for less than five years (3% at 9y and 1.7% at 2020), but 16.2% (at 9y) vs 23.3% had followed their faith for more than 5 years (but not all their life) ( Table 7a). Similar results were shown for their partners ( Table 7b).

The most notable difference between the 9 year and the 2020 sweep was the increase of professed non-believers in both the mothers (17.5% vs 29.8%) ( Table 7a) and partners (31.9% vs. 45.3%) ( Table 7b).

For those mothers who responded to the same questions at all four time points, a dramatic increase in those professing non-belief can be noted (from 6.6% antenatally to 19.6% in 2020). However, at 5 and 9 years the corresponding figures were similar at 10.0% and 9.8% respectively. There was a steady decrease in the numbers of mothers stating that they would appeal for help when in trouble ( Table 8a).

 For those mothers answering the type of religious beliefs they had had at each time point, those stating ‘none’ were fairly consistent at the first three points (range 25.6% to 27.3%) and then rose to 40.4% in 2020. A corresponding decrease was especially notable in those who stated they belonged to the Church of England (54.3% antenatally to 41.2% in 2020) ( Table 8a).

### Strengths and limitations of the data

The strengths of these data include the large sample size, with almost 7000 participants having data available from the 2020 sweep. The participants are broadly representative of the general population in the area, at the time of recruitment, in terms of sex, ethnicity, and socio-economic status ( Fraser *et al*., 2013). The extensive data on mediators, moderators, confounders, and physical and mental health outcomes to be collected over the next two years will facilitate huge amounts of research.

A key limitation of the data is the lack of ethnic diversity. At the time of enrolment, the county of Avon was mainly Caucasian, therefore there were too few Black, Asian and Minority Ethnic (BAME) participants (<6% in total) to allow for detailed analysis by ethnic background. A further limitation is that, as with all longitudinal studies there is increasing attrition over time. For these study parents, the loss is due mainly to mortality, change of address, as well as of reluctance to stay involved in the study. 

## Data availability

ALSPAC data access is through a system of managed open access. The steps below highlight how to apply for access to the data included in this paper and all other ALSPAC data. Note that Table 3 in this paper gives the variable numbers for the religion data.

1. Please read the ALSPAC access policy which describes the process of accessing the data and biological samples in detail, and outlines the costs associated with doing so.
2. You may also find it useful to browse our fully searchable research proposals database, which lists all research projects that have been approved since April 2011.
3. Please submit your research proposal for consideration by the ALSPAC Executive Committee using the online process. You will receive a response within 10 working days to advise you whether your proposal has been approved.
  If you have any questions about accessing data, please email: alspac-data@bristol.ac.uk (data) or bbl-info@bristol.ac.uk (samples).

The ALSPAC data management plan describes in detail the policy regarding data sharing, which is through a system of managed open access.

## Ethical approval and consent

Prior to commencement of the study, approval was sought from the ALSPAC Ethics and Law Committee and the Local Research Ethics Committees ( Birmingham, 2018). Informed consent for the use of data collected via questionnaires and clinics was obtained from participants following the recommendations of the ALSPAC Ethics and Law Committee at the time. Questionnaires were completed in the participants own home and return of the questionnaires was taken as continued consent for their data to be included in the study. Full details of the approvals obtained are available from the study ( http://www.bristol.ac.uk/alspac/researchers/research-ethics/). Study members have the right to withdraw their consent for elements of the study or from the study entirely at any time.
