# Supplementary material for: Parental spiritual and religious beliefs and behaviour data collected from the Avon Longitudinal Study of Parents and Children, 2020
Source: Wellcome Open Res. 2022 Nov 29;6:175. Originally published 2021 Jul 7. [Version 3] doi: 10.12688/wellcomeopenres.17010.3 (PMC9437533; doi:10.12688/wellcomeopenres.17010.3)
Supplement: Peer review history

# Peer review: Reviewer response for version 3

**DOI:** 10.21956/wellcomeopenres.20662.r53508

**Published:** 2022-12-07

**Authors:** Kendal Boyd

**Affiliations:** 1 Department of Psychology, Loma Linda University, Loma Linda, CA, USA

The Tables 8a and 8b are sufficient evidence for me to approve this data note.

Are sufficient details of methods and materials provided to allow replication by others?

Partly

Is the rationale for creating the dataset(s) clearly described?

Yes

Are the datasets clearly presented in a useable and accessible format?

Yes

Are the protocols appropriate and is the work technically sound?

Yes

Reviewer Expertise:

Psychology of religion; psychometrics

I confirm that I have read this submission and believe that I have an appropriate level of expertise to confirm that it is of an acceptable scientific standard.

# Peer review: Reviewer response for version 3

**DOI:** 10.21956/wellcomeopenres.20662.r53509

**Published:** 2022-12-02

**Authors:** Ralph L. Piedmont

**Affiliations:** 1 Center for Professional Studies, Timonium, MD, USA

I think things are very much improved, but did find two small issues. The first, is that the description to Table 6 still includes reference to p-values, which are no longer in that table. So the description needs to be adjusted. Second, in referring to information in table 8a, the authors note that there was a “dramatic” increase. This term needs to be deleted. Going from a 6% level to a 19% level is only that: a 13% increase. Without the use of any effect size index, it is not know the size of this change. So, delete “dramatic.” The other descriptions of percentage changes in the data are all accurate and well-done, not using any descriptors of magnitude change. Other than this, I believe my major concerns have been addressed.

Are sufficient details of methods and materials provided to allow replication by others?

Yes

Is the rationale for creating the dataset(s) clearly described?

Yes

Are the datasets clearly presented in a useable and accessible format?

Yes

Are the protocols appropriate and is the work technically sound?

Partly

Reviewer Expertise:

NA

I confirm that I have read this submission and believe that I have an appropriate level of expertise to confirm that it is of an acceptable scientific standard.

# Peer review: Reviewer response for version 2

**DOI:** 10.21956/wellcomeopenres.20304.r52180

**Published:** 2022-09-08

**Authors:** Ralph L. Piedmont

**Affiliations:** 1 Center for Professional Studies, Timonium, MD, USA

I am disappointed by the authors' response to my review. The authors responded to my initial comments saying that this paper was just a descriptive study and not a research report. Yet, they are conducing statistical analyses of the data to show that there are significant changes in response patterns over time. 

 My problem with the statistics is that all the p-levels are very low, namely because the sample sizes are very large. Just giving p-values may lead readers to assume that the observed effects were very strong, when, in fact, they are small to moderate. I noted in my initial comments that when providing statistical results one needs to provide all relevant data for that analysis. In this report, only p-levels are given. The authors should provide the statistical analysis they used (from their comments it seems they used a chi square test), associated degrees of freedom, the obtained value and its associated p-level. In this manner, readers have sufficient information to accurately interpret the findings. 

 The paper analyzes and presents data from established studies with the intent of deriving inferences from that data. The authors fail to provide readers with appropriate cautions, not only about the data analyses but the scales as well. There are problems with these measures, but they must let the reader know of these limitations. Good form and appropriate application of professional standards is essential for any good paper. To justify this by referring to this paper as "descriptive" is unacceptable.

Are sufficient details of methods and materials provided to allow replication by others?

Yes

Is the rationale for creating the dataset(s) clearly described?

Yes

Are the datasets clearly presented in a useable and accessible format?

Yes

Are the protocols appropriate and is the work technically sound?

Partly

Reviewer Expertise:

I have been involved in this field for over 25 years; I have served as the founding editor of the APA journal, Psychology of Religion and Spirituality, editor of Research in the Social Scientific Study of Religion. I have published numerous articles on this topic, with special emphasis on measurement/scale development, clinical predictiveness of R/S constructs, and cross-cultural research.

I confirm that I have read this submission and believe that I have an appropriate level of expertise to confirm that it is of an acceptable scientific standard, however I have significant reservations, as outlined above.

# Response

**Published:** 2022-11-22

**Authors:** Yasmin Iles-Caven

**Affiliations:** University of Bristol, UK

We are sorry you were disappointed with our response. We hope the below will clarify our meanings.

 We probably did not describe clearly the nature of a Data Note as described by the Wellcome Open Research journal itself: “Data Notes are brief descriptions of scientific datasets that promote the potential reuse of research data and include details of why and how the data were created; they do not include any analyses or conclusions.” They go on to point out the likely benefits of publishing Data Notes as sharing research datasets in a discoverable, useable and reproducible way, and fostering new collaborations across disciplines.

 In retrospect, given this we are convinced that we should not have carried out any analyses or used P values at all. We have therefore removed them from Table 6, and from the text.

 Furthermore, in answer to the criticism concerning the questions used, we have added to the ‘Limitations’ section in the Discussion the sentence: ‘We acknowledge that the RSBB measures chosen for the study are not those that are approved by all. We asked advice of a number of experts in the field, but not all responded. The data such that it is are described here so that any researchers who would like to use them will see what is available and how to access it."

# Peer review: Reviewer response for version 2

**DOI:** 10.21956/wellcomeopenres.20304.r52181

**Published:** 2022-09-02

**Authors:** Kendal Boyd

**Affiliations:** 1 Department of Psychology, Loma Linda University, Loma Linda, CA, USA

The authors have addressed some of the concerns over their interpretation of these data, however, they still have not addressed the issue of socioeconomic bias in those who continue to remain in the study. There is some evidence that socioeconomic status is inversely related to religiosity, at least in the United States (Scheiman, 2010), but also globally (Rachmatullah, Ha, & Park, 2019).

 Although this is countered by their reference that suggests that the study's religious respondents were more likely to continue in the study, it still should be noted that the findings of decreased religiosity over time could, in part, be due to the remaining sample's increased socioeconomic status.

 The bias for the dataset over time to be of a higher socioeconomic status seems to be an important consideration for anyone using it for subsequent analyses, in particular when making interpretations about the religious trends of the population in England.

Are sufficient details of methods and materials provided to allow replication by others?

Partly

Is the rationale for creating the dataset(s) clearly described?

Yes

Are the datasets clearly presented in a useable and accessible format?

Yes

Are the protocols appropriate and is the work technically sound?

Yes

Reviewer Expertise:

Psychology of religion; psychometrics

I confirm that I have read this submission and believe that I have an appropriate level of expertise to confirm that it is of an acceptable scientific standard, however I have significant reservations, as outlined above.

## References

[1] : Socioeconomic Status and Beliefs about God's Influence in Everyday Life. *Sociology of Religion* .2010;71(1) : 10.1093/socrel/srq004 25-51 10.1093/socrel/srq004

[2] : Relations among education, religiosity and socioeconomic variables. *South African Journal of Education* .2019;39(1) : 10.15700/saje.v39n1a1611 1-13 10.15700/saje.v39n1a1611

# Response

**Published:** 2022-11-22

**Authors:** Yasmin Iles-Caven

**Affiliations:** University of Bristol, UK

We agree that there are various ways in which to interpret the trends in prevalence. In order to address the possibility that the changes maybe due to social or other biases in the attrition rate we presented the data for the adults who had answered all four questionnaires in Tables 8a and 8b. They show a drop in prevalence of religiosity even within the same individuals.

 This, we feel is one relatively simple way of answering the question as to the likelihood of bias from differential follow-up in comparing results from one data sweep to the next.

# Peer review: Reviewer response for version 2

**DOI:** 10.21956/wellcomeopenres.20304.r52179

**Published:** 2022-09-01

**Authors:** Ellen Idler

**Affiliations:** 1 Department of Sociology and the Rollins School of Public Health, Emory University, Atlanta, GA, USA

The framing of the front end of the paper is improved, my biggest concern. I approve.

Are sufficient details of methods and materials provided to allow replication by others?

Yes

Is the rationale for creating the dataset(s) clearly described?

Yes

Are the datasets clearly presented in a useable and accessible format?

Yes

Are the protocols appropriate and is the work technically sound?

Partly

Reviewer Expertise:

NA

I confirm that I have read this submission and believe that I have an appropriate level of expertise to confirm that it is of an acceptable scientific standard.

# Peer review: Reviewer response for version 1

**DOI:** 10.21956/wellcomeopenres.18785.r51348

**Published:** 2022-08-17

**Authors:** Kendal Boyd

**Affiliations:** 1 Department of Psychology, Loma Linda University, Loma Linda, CA, USA

The study describes the decline in the religiosity of parents and partners in the Bristol, England, area over time, and the longitudinal correlates of this, which will be a helpful empirical contribution. 

 One methodological issue is that, due to attrition, the sample became older and more educated over time in that the younger and less-educated respondents were more likely to drop out of the study. This is a systematic bias and doesn't appear to be random.

 A related issue is the replacement of missing data with imputation; however, the study does not specify how this will be accomplished. 

 It will be important for the imputation procedure to take into account the non-random pattern of missing data, and for this to be described in detail so it could be replicated by other researchers. Further, the limitations of the scientific generalization of the findings will need to be made clear. 

 Also, as the authors note, the religious traditions represented are primarily Christian, and again caution will have to be taken in generalizing these results to non-Christian religions.

Are sufficient details of methods and materials provided to allow replication by others?

Partly

Is the rationale for creating the dataset(s) clearly described?

Yes

Are the datasets clearly presented in a useable and accessible format?

Yes

Are the protocols appropriate and is the work technically sound?

Yes

Reviewer Expertise:

Psychology of religion; psychometrics

I confirm that I have read this submission and believe that I have an appropriate level of expertise to confirm that it is of an acceptable scientific standard, however I have significant reservations, as outlined above.

# Response

**Published:** 2022-08-23

**Authors:** Yasmin Iles-Caven

**Affiliations:** University of Bristol, UK

We thank the reviewer for their helpful comments.

**re Attrition**: We have added two sentences to the Strengths & Limitations section (as reviewer 1 had also highlighted attrition). I am repeating our answer to Rev 1:  Attrition is of major concern in ALSPAC and known to be non-random and likely to result in selection bias (see e.g. Taylor et al 2018; Cornish et al 2021). It has been higher in those mothers from lower socioeconomic backgrounds, with complications in the index pregnancy and those with a lack of social support. More recent data collection shows a positive bias in participation towards older mothers, those with co-habiting partners in pregnancy, educated to degree level, higher socioeconomic status (Fraser et al., 2013) and more likely to attend a place of worship at least monthly (Morgan et al., 2022). The latter paper, in particular, discusses the ways in which these biases may be addressed in future analyses.

 Cornish RP, Macleod J, Boyd A, Tilling K. Factors associated with participation over time in the Avon Longitudinal Study of Parents and Children: a study using linked education and primary care data. International journal of epidemiology. 2021 Feb;50(1):293-302.

 Morgan J, Halstead I, Northstone K, Major-Smith D.  Religious/spiritual beliefs and behaviours and study participation in a prospective cohort study (ALSPAC) in Southwest England.  Wellcome Open Research 2022; 7:186.

 Taylor AE, Jones HJ, Sallis H, Euesden J, Stergiakouli E, Davies NM, Zammit S, Lawlor DA, Munafò MR, Davey Smith G, Tilling K. Exploring the association of genetic factors with participation in the Avon Longitudinal Study of Parents and Children. International journal of epidemiology. 2018 Aug 1;47(4):1207-16.

**A related issue is the replacement of missing data with imputation; however, the study does not specify how this will be accomplished**. A related paper [Morgan et al., 2022] has discussed this in regard to the RSBB data in ALSPAC as follows: “Religious attendance appears to be ‘missing not at random’ (MNAR, i.e., continued participation may depend on religious attendance). As such, when using religious attendance as an exposure or outcome, there is a risk of potential selection bias. Additional sensitivity analyses—such as not at random multiple imputation (Lee et al., 2021; Tompsett et al., 2018), simulations (Millard et al., 2021) or Bayesian approaches to model selection (Du et al., 2022)—may be required to explore the extent of selection bias and whether this may impact the subsequent conclusions drawn (Griffith et al., 2020; Smith, 2020). Additionally, other aspects of RSBB—belief in God/a divine power and religious affiliation—were frequently associated with continued participation in unadjusted analyses, but these associations were often attenuated to null after adjusting for potential confounders. This suggests that these RSBB factors may be ‘missing at random’ (MAR, i.e., adjusting for confounders included here, religious belief and affiliation are not associated with selection). These RSBB factors may not therefore be MNAR, and so are at less risk of causing selection bias; however, to avoid these RSBB variables resulting in selection bias, these demographic and socioeconomic confounders would also need to be controlled for in subsequent analyses. Although some studies have suggested that association with participation would have to be fairly severe for the subsequent bias to cause concern (Cornish et al., 2021; Pizzi et al., 2011), this needs to be explored on a case-by-case basis. It is hoped that highlighting the potential for selection bias in ALSPAC’s RSBB data will inform future users of the resource.“

 We have therefore added a couple of sentences to this effect in our revised version of the Data Note (to the end of Strengths and Difficulties). Morgan et al., 2022 reference has been added to the reference list.

# Peer review: Reviewer response for version 1

**DOI:** 10.21956/wellcomeopenres.18785.r51377

**Published:** 2022-08-16

**Authors:** Ralph L. Piedmont

**Affiliations:** 1 Center for Professional Studies, Timonium, MD, USA

The purpose of this report was to provide additional assessments on a longitudinal sample regarding issues of religious involvement, spirituality, faith development, and family factors that influence religious/spiritual development in children. This paper provides a fourth evaluation of this sample. Data were collected that examined religious involvements of parents, their partners, on their children. Longitudinal data sets like this one are important for charting generational changes in key variables. While there is value here, with the potential for more to come, there are some issues that need to be addressed.

  

1. The authors tend to make some very strong statements about the value of longitudinal designs over cross-sectional ones. For example, the authors noted, “Any study designed to identify whether some aspect of religious/spiritual beliefs or behaviours (RSBB) affects physical or mental health must analyse data longitudinally.” This is false. Longitudinal designs have no advantage over cross-sectional ones in terms of evaluation causality. This paper (Piedmont, Fox, and Toscano 2020) ^1^ is an example of a cross-sectional study, using SEM analyses to identify the causal precedence of spiritual variables on mental health functioning. While not a longitudinal study, it nonetheless is able to evaluate the relative merits of different causal models. Only a research design can examine causality, whether one is conducting a cross-sectional or longitudinal study. Without an experimental manipulation and random assignment of subjects, any study becomes correlational in nature. Such sweeping statements need to be avoided. This longitudinal study has nothing to contribute to the examination of any causal effects, it is merely descriptive in nature. I am referencing some papers that do examine causal issues using cross-sectional data.
2. I am completely underwhelmed by the selection of measures employed in this study. Many of the study’s own measures are simply single item scales that require only a “yes/no” response. Such items are quite useless because they contain so much error. It is unclear what a “yes” or “no” answer really means because no normative data is given. If someone says that they are spiritual, how spiritual are they? Do we just lump all the “yes” responders together in a single group? Not very informative. Further, without controlling for potential mediators, in this case factors like economic status, ethnicity, and related constructs, it is not clear what is uniquely important about responses to these items. It was noted that gender was a factor influencing responses, but no effort was made at explaining why this is. No effort was made to determine what influence gender had on score changes over time. This would be important to know. What else is going on in the lives of these people that may be influencing their responses. England has been going through lots of economic and political upheaval over the course of this study (e.g., Brexit, the new “leveling up” policies, etc.), all of which may impact these scores. Some control for potential mediators and moderators of responses needs to be given, especially when only single items are used. More on this below.
3. The other scales used in this study are also quite problematic. The DUREL scale is widely used in medical research, but because all the items are positively phrased, the scale is contaminated by acquiescence bias. One reason why this scale correlates well with other religious/spiritual (R/S) scales is because many of those are similarly biased (like those on the BMMRS scale). This issue needs to be acknowledged as a relevant limitation to the study. The other scale used, the Fetzer BMMRS is noted by the authors as being,  "from the well-validated Fetzer Brief Multi-Dimensional Measure of Religiosity/Spirituality.” I am not aware that the BMMRS is well-validated. The measure is NOT a scale in any sense of the term. It is merely a collection of approximately 32 items taken across 12 different constructs. There is no scalar information about this instrument and is best considered an epidemiological screener. Without any normative information about responses to these items, it is difficult to interpret their meaning. However, this is a fixable issue whereas the manual to this instrument does give mean level data for each item. This information can be used as a comparison to the current data. Finally, it is not clear to my why such out of date scales are used, like the Intrinsic-Extrinsic Religiosity Scale? This is a very old scale with problems to it. There are so many newer, and better scale available that can be more useful, like the Assessment of Spirituality and Religious Sentiments (ASPIRES) scale or the Numinous Motivation inventory (see www.centerforprofessionalstudies.com for information about both). One the one hand, continuity is important in a longitudinal study, but it would be helpful if some better instruments are also included.
4. The data are just descriptive in nature, and clearly show a decrease in R/S salience for the sample over time. Why this is, is not discussed or examined. Similar data trends have been found by other organizations, like the Pew Foundation in the US which has documented the rise of the non-affiliated. It would be helpful conceptually for readers if connections with these other studies are made. Where data analyses are conducted no real statistical information is given. First, providing p-values as way of demonstrating effect is problematic; p-values are NOT measures of effect size and should never be used as a means of presenting results. I would like to know, what exact statistical tests were conducted in comparing mothers and their partners and what the observed values for those tests were. In this manner, readers can get a better sense of the magnitude of effects being presented. Given the very large sample sizes noted here, it is completely expected that any statistical test will generate a very small p-value. Even small effects can generate small p-values. As we know, when a two-tailed test is used, as sample size increases the probability of rejecting the null moves to 1.0. The authors also need to comment on the actual effect sizes noted in their analyses. How much of a change is really going on here?  I would suggest that the authors drop the p-values and give appropriate effect size estimates.
5. I think the data here have great potential. I am looking forward to seeing more results from this data set. I hope that the authors will consider providing longitudinal analyses that will examine the causal precedence of the relevant variables in the study. This can be done nicely either with a cross-lagged panel design or using structural equation modeling. Hopefully, appropriate mediational analyses will be conducted. 

Are sufficient details of methods and materials provided to allow replication by others?

Yes

Is the rationale for creating the dataset(s) clearly described?

Yes

Are the datasets clearly presented in a useable and accessible format?

Yes

Are the protocols appropriate and is the work technically sound?

Partly

Reviewer Expertise:

I have been involved in this field for over 25 years; I have served as the founding editor of the APA journal, Psychology of Religion and Spirituality, editor of Research in the Social Scientific Study of Religion. I have published numerous articles on this topic, with special emphasis on measurement/scale development, clinical predictiveness of R/S constructs, and cross-cultural research.

I confirm that I have read this submission and believe that I have an appropriate level of expertise to confirm that it is of an acceptable scientific standard, however I have significant reservations, as outlined above.

## References

[1] : Spiritual Crisis as a Unique Causal Predictor of Emotional and Characterological Impairment in Atheists and Agnostics: Numinous Motivations as Universal Psychological Qualities. *Religions* .2020;11(11) : 10.3390/rel11110551 10.3390/rel11110551

# Response

**Published:** 2022-08-23

**Authors:** Yasmin Iles-Caven

**Affiliations:** University of Bristol, UK

We thank you for your helpful and insightful comments on this paper.

**Your point 1**.  Firstly, this is purely a descriptive paper (data note) of the data available on RSBB collected in 2020 and the ability to link this to a rich phenotypic dataset collected over a 30-year period including RSBB data collected antenatally and at 5 and 9 years post-partum.  It is not a research article.

 We apologise for our sweeping statement concerning longitudinal studies. We agree cross-sectional studies have their value, but in order to examine trajectories of poor mental and/or physical health and whether RSBB has any influence or causality on the (non-) development of disease, longitudinal studies are required (e.g. non-genetic inheritance via grandmaternal smoking in pregnancy and RSBB in granddaughters (Golding et al., 2022); or RSBB influences on the initiation or giving up of habits such as alcohol, smoking and drug use as well as aspects of diet, such factors may be on the causal pathway between RSBB and aspects of mental or physical health. Such features are most easily interpreted when it is clear as to the sequence of exposures, mediators and outcomes.   

 Golding J, Gregory S, Northstone K, Pembrey M, Ellis G, Watkins S, Iles-Caven Y, Suderman M. Possible transgenerational associations between grandparents’ childhood exposures and religious belief in their granddaughters: a longitudinal cohort study. Wellcome Open Research. 2022 Aug 12;7(213):213.

**Selection of measures**. The RSBB data collected within ALSPAC questionnaires only comprises one section among many others including socioeconomic status, health, measures of anxiety and depression, personality, social interactions, locus of control, and traumatic experiences.  We are therefore not limited in our exploration of RSBB in itself.

 To repeat, this is a description of RSBB data collected in this wave. Data Notes, as defined by Wellcome Open Research do not involve detailed analyses. They are meant to describe the relevant data available and the ways in which the interested researcher may access it.

**Your point 2**.  We have omitted the phrase ‘well-validated’ from the paper.  We are sorry you have been less than enthusiastic about some of the measures used. We relied on recommendations from experts in the field.  This paper merely describes what is available to the interested researcher.

**Your point 3 Statistical analyses**. We have compared proportions using chi-squared, and quoted the corresponding P value. We do not claim that this is equivalent to an effect size. Rather it is there as an indicator of a difference that may not be explained by chance. The data provided in the tables is such that the interested researcher can calculate unadjusted effect sizes if required.  Again, we reiterate this is purely a descriptive paper.  ALSPAC welcomes collaboration (see data availability section).

**Your point 4**. There are a number of researchers working with these data, but more are always welcomed. Because of the broad wealth of data collected by ALSPAC, there are a large number of questions that we hope that interested researchers may address.

# Peer review: Reviewer response for version 1

**DOI:** 10.21956/wellcomeopenres.18785.r44836

**Published:** 2022-03-25

**Authors:** Ellen Idler

**Affiliations:** 1 Department of Sociology and the Rollins School of Public Health, Emory University, Atlanta, GA, USA

This manuscript presents descriptive data from a 2020 (4 ^th^ wave) follow-up of a long-running cohort study of parents and children born to them in Bristol, England in the early 1990s. The children are now in their late 20s. The focus of the data presented is on a large number of measures of religiousness and spirituality, some of which (but not all) had been asked in previous waves. The major take-away is the decline in religious and spiritual beliefs and behaviours reported by the participants during the period of the study. The manuscript is well-written and clear for the most part.

1. Table 1 usefully compares the 2000-2001 stated religious affiliation of sample mothers and partners with both the local Bristol population and the population of England/Wales at the time. The text notes that the study sample was more likely to state that they had no religion than the national census but at the same time they were considerably more likely to state that they were Christian than the surrounding local area census shows. Given the attrition from the sample (see #2), it would be important to continue benchmarking the results against whatever local data are available.
2. The sample is dramatically reduced in size by the 2020 follow-up. This is acknowledged briefly in the limitations section of the discussion, but I would say that it is a much more serious issue. I do not see sample sizes for the 2 ^nd^ and 3 ^rd^ waves of data collection, but with the Ns that are given for T1 and T4, it appears that only 37% of the mothers (4525/12,243) and 24.5% (2349/9583) of the partners who began the study in 1991/92 provided data in 2020. Table 2 usefully tells us that a considerable amount of selection has occurred such that 2020 sample mothers were older, better educated, more likely to have been living with their partner, and more likely the mothers of girls compared with those who attrited from the study. This is really a huge amount of loss, and it calls for some procedures, perhaps Heckman correction models or other ways of attending to the ways in which the sample is different from the population from which it came. Without this, and a much more major acknowledgement of the magnitude of the attrition, the implications of the language in the text and abstract, of a change over time in RSBB is quite misleading.
3. Another area of concern is the meaning of these measures of religious belief and practice in the context of an extremely secular environment. What does it mean to say that one has brought up one’s child in one’s current faith (2/3 of parents say they have done this) when half or more than half “never “go to a place of worship and consider themselves “not at all religious” and fewer than half say they believe in God or some divine power? I could go on with more examples of the seeming contradictions here. Some qualitative interviews might help uncover the meaning of these responses.

Are sufficient details of methods and materials provided to allow replication by others?

Yes

Is the rationale for creating the dataset(s) clearly described?

Yes

Are the datasets clearly presented in a useable and accessible format?

Yes

Are the protocols appropriate and is the work technically sound?

Partly

Reviewer Expertise:

My areas of research include social factors in health, aging and perceptions of health, religion as a social determinant of health. I am a sociologist with training in epidemiology.

I confirm that I have read this submission and believe that I have an appropriate level of expertise to confirm that it is of an acceptable scientific standard, however I have significant reservations, as outlined above.

# Response

**Published:** 2022-08-23

**Authors:** Yasmin Iles-Caven

**Affiliations:** University of Bristol, UK

**The authors thank the reviewer for their helpful and insightful comments, which we endeavour to address:**

**1. Thank you for this suggestion, we aim to add census data when available to our blog: https://ahrp.blogs.bristol.ac.uk/**

**2. Attrition**

 Attrition is of major concern in ALSPAC and known to be non-random and likely to result in selection bias (see e.g. Taylor et al., 2018; Cornish et al., 2021). It has been higher in those mothers from lower socioeconomic backgrounds, with complications in the index pregnancy and those with a lack of social support.  More recent data collection shows a positive bias in participation towards older mothers, those with co-habiting partners in pregnancy, educated to degree level, higher socioeconomic status (Fraser et al., 2013) and more likely to attend a place of worship at least monthly (Morgan et al., 2022). The latter paper, in particular, discusses the ways in which these biases may be addressed in future analyses.

 Cornish RP, Macleod J, Boyd A, Tilling K. Factors associated with participation over time in the Avon Longitudinal Study of Parents and Children: a study using linked education and primary care data. International journal of epidemiology. 2021 Feb;50(1):293-302.

 Morgan J, Halstead I, Northstone K, Major-Smith D.  Religious/spiritual beliefs and behaviours and study participation in a prospective cohort study (ALSPAC) in Southwest England.  Wellcome Open Research 2022; 7:186.

 Taylor AE, Jones HJ, Sallis H, Euesden J, Stergiakouli E, Davies NM, Zammit S, Lawlor DA, Munafò MR, Davey Smith G, Tilling K. Exploring the association of genetic factors with participation in the Avon Longitudinal Study of Parents and Children. International journal of epidemiology. 2018 Aug 1;47(4):1207-16.

**3. Meaning of measures**

 Format of all questions have been through extensive ethical and participant vetting in order to be inclusive. To this end (see Table 3), the actual question asked (C8). Did you bring your (child)ren up in your current faith/belief ( **including none**)? Yes, this faith/No.  If no, what faith did you bring your children up in, **if any** (describe)?  This refers back to question C5. What sort of religious faith would you say you had? Options included ‘None’. Therefore those responding ‘yes’ to C8 include the ‘nones’. Text responses have yet to be coded which may elucidate further.
